# Cultivating Functional Natural Killer Cells from Mobilized Hematopoietic Stem Cells in Heavily Pretreated Hematologic Malignancies

**DOI:** 10.3390/ijms27135836

**Published:** 2026-06-28

**Authors:** Suppanut Komjakraphan, Poonnattha Anantasaeree, Kajornkiat Maneechai, Panarat Noiperm, Jakrawadee Julamanee

**Affiliations:** 1 Hematology Unit, Division of Internal Medicine, Faculty of Medicine, Prince of Songkla University, Hat Yai, Songkhla 90110, Thailand; suppanut.k@psu.ac.th (S.K.); 6510310066@email.psu.ac.th (P.A.); kajornkiat.m@psu.ac.th (K.M.); panarat.n@psu.ac.th (P.N.); 2 Stem Cell Laboratory, Stem Cell Transplantation and Cellular Therapy Excellence Center, Songklanagarind Hospital, Faculty of Medicine, Prince of Songkla University, Hat Yai, Songkhla 90110, Thailand; 3 Thailand Hub of Talents in Cancer Immunotherapy (TTCI), Bangkok 10330, Thailand

**Keywords:** hematopoietic stem cell, natural killer, cancer, immunotherapy

## Abstract

CD19 chimeric antigen receptor (CAR) T cells have demonstrated promising outcomes in B-cell malignancies. However, using pretreated autologous T cells currently faces limitations, including compromised T-cell fitness and the challenge of manufacturing sufficient cell numbers for treatment. Consequently, natural killer (NK) cells have emerged as an alternative due to their natural ability to mediate cytotoxicity and their favorable safety profile. This study aims to generate patient autologous hematopoietic stem cell-derived NK (HSC-NK) cells and assess their therapeutic potential compared to peripheral blood NK (PB-NK) cells. We successfully cultivated HSC-NK under a 28-day, two-step differentiation and expansion protocol, achieving a cumulative 290-fold expansion using optimized memory-like cytokines and feeder cell stimulation. The expanded HSC-NK cells demonstrated a distinct phenotype (CD56^+^CD16^low^), representing an immature differentiation state, characterized by a lower expression of inhibitory receptors (NKG2A, KIR2DL, and CD94) and the exhaustion markers (LAG3, PD-1, TIM-3, and CTLA-4) compared to PB-NK cells. Prominent expression of CD62L, alongside sustained expression of CD69 and CD107a, was observed, translating into NK cell proliferation, activation, and cytotoxicity against cancer cells comparable to PB-NK cells. In conclusion, generating HSC-NKs is feasible while preserving essential NK cell phenotypes and activities. Our findings emphasize the potential of HSCs as an alternative NK cell source for cancer immunotherapy.

## 1. Introduction

Adoptive cell therapy, particularly CD19-targeted chimeric antigen receptor T-cell (CAR-T) therapy, has revolutionized the treatment of relapsed/refractory (R/R) B-cell lymphomas [1,2,3]. Despite impressive response rates, various unmet needs still persist in current autologous CAR-T cell therapies, such as ineffective T-cell function in heavily pretreated donors who exhibit exhausted T-cell phenotypes or inadequate T-cell numbers for therapeutic purposes [4,5,6]. Moreover, severe treatment-related toxicities, such as cytokine release syndrome (CRS) and immune effector cell–associated neurotoxicity syndrome (ICANS), frequently occur in patients treated with CAR-T cells [7,8]. To overcome these issues, alternative immune cells—particularly innate immune cells that function independently of the major histocompatibility complex (MHC)—are being explored as potential cancer therapies.

Natural killer (NK) cells are lymphocytes that function at the interface between innate and adaptive immunity. NK cells operate without prior sensitization, specific antigen recognition, or clonal expansion; they naturally kill transformed, infected, and malignant cells without MHC restriction. Various studies have reported on the safety and efficacy of NK cell therapy in hematopoietic cell transplantation and adoptive NK cell transfer in clinical trials [9,10,11,12]. NK cells express a wide range of receptors that mediate activating and inhibitory signals, the integration of which regulates NK cell functions, including proliferation, cytotoxic activity, and cytokine secretion [13]. Their function is largely influenced by human leukocyte antigen (HLA) compatibility, specifically interactions between killer immunoglobulin-like receptors (KIRs) on NK cells and HLA molecules on target cells [14].

NK cells are categorized into two subtypes based on their CD56 and CD16 expression. CD56^dim^CD16^+^ NK cells constitute the majority of circulating NK cells and are highly cytotoxic, exerting their function through perforin- and granzyme-mediated lysis and antibody-dependent cellular cytotoxicity (ADCC) via CD16 (FcγRIII) [15]. In contrast, NK cells derived from HSCs are predominantly CD56^bright^CD16^low^; these cells exhibit low cytotoxicity but high cytokine production, secreting interleukin-10, interferon-gamma (IFN-γ) and tumor necrosis factor-alpha and beta (TNF-α/β), which enhance immune modulation and promote hematopoietic recovery [16]. While peripheral blood NK cells (PB-NKs) are more effective for direct tumor killing, hematopoietic stem cell-derived NK cells (HSC-NKs) play a crucial role in immune regulation, possess greater proliferative capacity, and can be efficiently expanded in vivo.

Conventionally, NK cells are sourced from peripheral blood (PB), umbilical cord blood (UCB), or induced pluripotent stem cells (iPSCs). Among these, PB-NK cells are easily accessible and naturally cytotoxic, making them a preferred choice for immediate therapeutic use. However, they have limited ex vivo expansion capacity, which restricts their large-scale clinical application [17]. Umbilical cord blood NK (UCB-NK) cells offer an allogeneic source with a reduced risk of graft-versus-host disease (GVHD), but their clinical utility is constrained by limited resource availability [18]. Induced pluripotent stem cell-derived NK (iPSC-NK) cells provide an unlimited, renewable NK cell source and are highly amenable to genetic modifications; nevertheless, their differentiation protocols are complex, and large-scale production remains a challenge [19]. In this context, HSC-NK cells emerge as a promising alternative immune cell source, as they can differentiate into functional NK cells with enhanced proliferative potential, making them ideal candidates for chimeric antigen receptor (CAR)-engineering [20]. Compared to CAR-T cells, CAR-engineered NK (CAR-NK) cells offer several advantages, including a lower risk of CRS, a minimal risk of GVHD, enhanced tumor recognition via both CAR-dependent and innate receptor-mediated mechanisms, and the potential for “off-the-shelf” availability due to their compatibility across multiple recipients [21].

To circumvent the limited cell supply inherent in conventional sources, utilizing heavily pretreated autologous patient samples in this study served as a rigorous stress test model. By demonstrating the robustness and expansion capacity of our protocol under these suboptimal conditions, we highlight its potential utility for future large-scale manufacturing. Subsequently, we evaluated the therapeutic potential of these generated HSC-NK cells by comparing their immunophenotypic and functional characteristics to those of conventional PB-NK cells, thereby establishing HSCs as a highly scalable and viable alternative immune cell source for engineered cancer immunotherapy.

## 2. Results

### 2.1. Donor Clinical Characteristics

Peripheral blood hematopoietic stem cell (PBSC) samples were obtained from five patients who underwent autologous hematopoietic stem cell transplantation (HSCT) for multiple myeloma (MM; *n* = 3), diffuse large B-cell lymphoma (DLBCL; *n* = 1), and Hodgkin lymphoma (HL; *n* = 1). The median age was 65 years (range: 37–66), and four patients were male. All patients had received prior chemotherapy with a median of one line (range: 1–3), followed by a high-dose cytarabine/granulocyte colony-stimulating factor (G-CSF)-based mobilization regimen (Table S1).

### 2.2. Efficient HSC-NK Cell Generation from Patient PBSC Products

**Figure 1:**
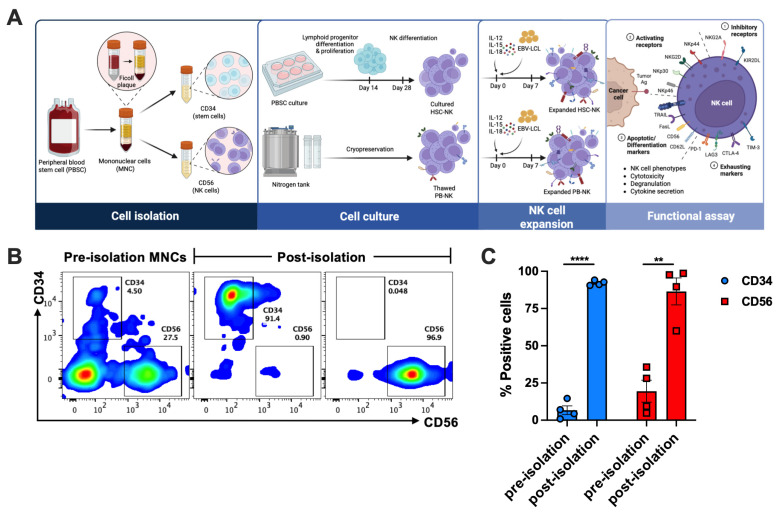
Peripheral blood hematopoietic stem cell (PBSC) and natural killer cell (PB-NK) isolation and assays. (**A**) Illustration of hematopoietic stem cell (HSC)-derived NK or PB-NK cell generation, expansion, and functional assays. (**B**) Representative flow cytometry plots pre- and post-isolation: mononuclear cells (MNCs) (left), CD34^+^ cells (middle), and CD56^+^ cells (right). (**C**) Percentages of CD34^+^ and CD56^+^ cells pre- and post-isolation. Data are pooled from four different donors and presented as the mean ± SEM. A Student’s *t*-test was used for (**C**); ** *p* < 0.01, **** *p* < 0.0001.

To generate HSC-NK cells from PBSC products, we initially isolated mononuclear cells (MNCs) using density gradient centrifugation, followed by the enrichment of CD34^+^ and CD56^+^ cells via immunomagnetic selection (Figure 1A). The percentages of pre-isolated CD34^+^ and CD56^+^ cells were 6.7% (range: 0.87–14.60%) and 19.49% (range: 4.76–35.70%), respectively. Post-isolation, the purity significantly increased to 92.12% (range: 90.50–94.10%) for CD34^+^ and 86.47% (range: 60.00–98.64%) for CD56^+^ cells (Figure 1B,C).

**Figure 2:**
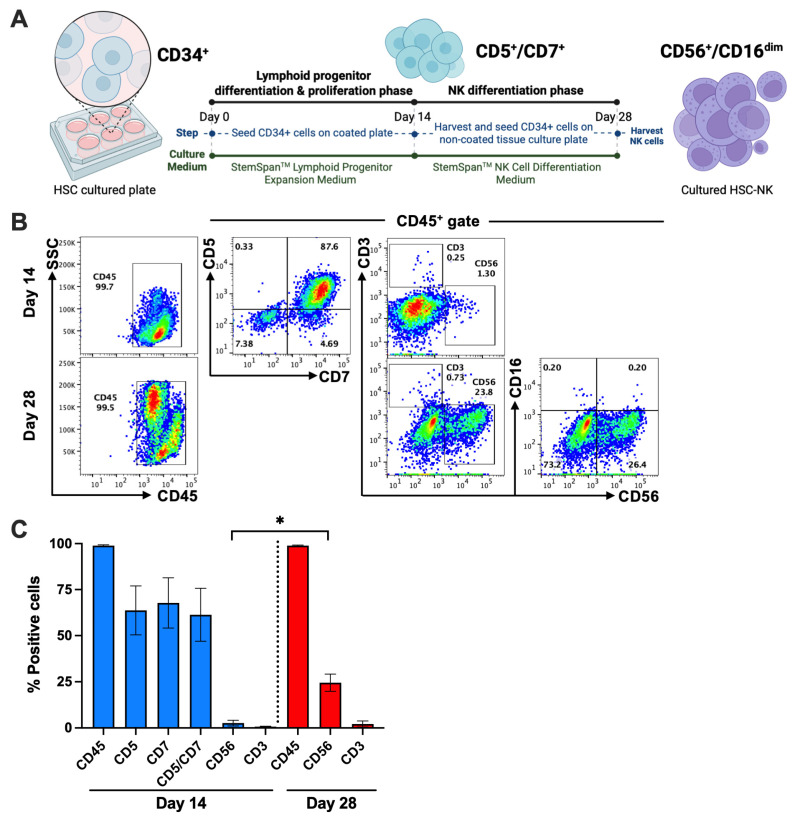
Hematopoietic stem cell (HSC)-derived natural killer (NK) generation. (**A**) Schematic of the HSC-NK generation protocol, consisting of two steps: (1) lymphoid progenitor differentiation and proliferation phase (days 0–14) and (2) NK cell differentiation phase (days 14–28). (**B**) Representative flow cytometry plots of HSC-derived lymphoid progenitor cells (day 14; upper panel) and HSC-derived NK cells (day 28; lower panel). (**C**) Percentages of HSC differentiation subsets after the lymphoid differentiation phase (day 14) and NK cell differentiation phase (day 28). Data are pooled from three different donors and presented as the mean ± SEM. A Student’s *t*-test was used for (**C**); * *p* < 0.05.

The enriched CD34^+^ cells were then cultivated for 28 days to induce differentiation into mature NK cells through a two-phase culture system: (1) a lymphoid progenitor differentiation and proliferation phase (days 0–14); and (2) an NK cell differentiation phase (days 14–28) (Figure 2A). During the first 14 days, HSCs were maintained in a lymphoid progenitor expansion medium supplemented with stem cell factor (SCF), thrombopoietin (TPO), FMS-like tyrosine kinase 3 ligand (Flt3L), and interleukin (IL)-7 to promote lymphoid commitment. The harvested cells were assessed for early lymphoid progenitor markers, and expressed CD5^+^ (63.7%), CD7^+^ (67.7%), and CD5^+^/CD7^+^ (61.3%). In contrast, CD56+ and CD3^+^ cells were minimally detected, at only 2.64% and 0.7%, respectively, during this phase (Figure 2B,C). Following the NK cell differentiation phase, a significant increase in CD56^+^ cells (24.53%) was detected by day 28, accompanied by a lack of CD3 expression, indicating differentiation toward the NK cell phenotype. The low expression of CD16—which facilitates ADCC—was detected within the CD56^+^ population, suggesting an immature state of the HSC-derived NK cells (Figure 2B). In conclusion, generating HSC-NK cells from autologous patient PBSC products was feasible using this two-phase culture system.

### 2.3. HSC-NK Cells Display Distinctive Phenotypic Signatures Compared to PB-NK Cells

**Figure 3:**
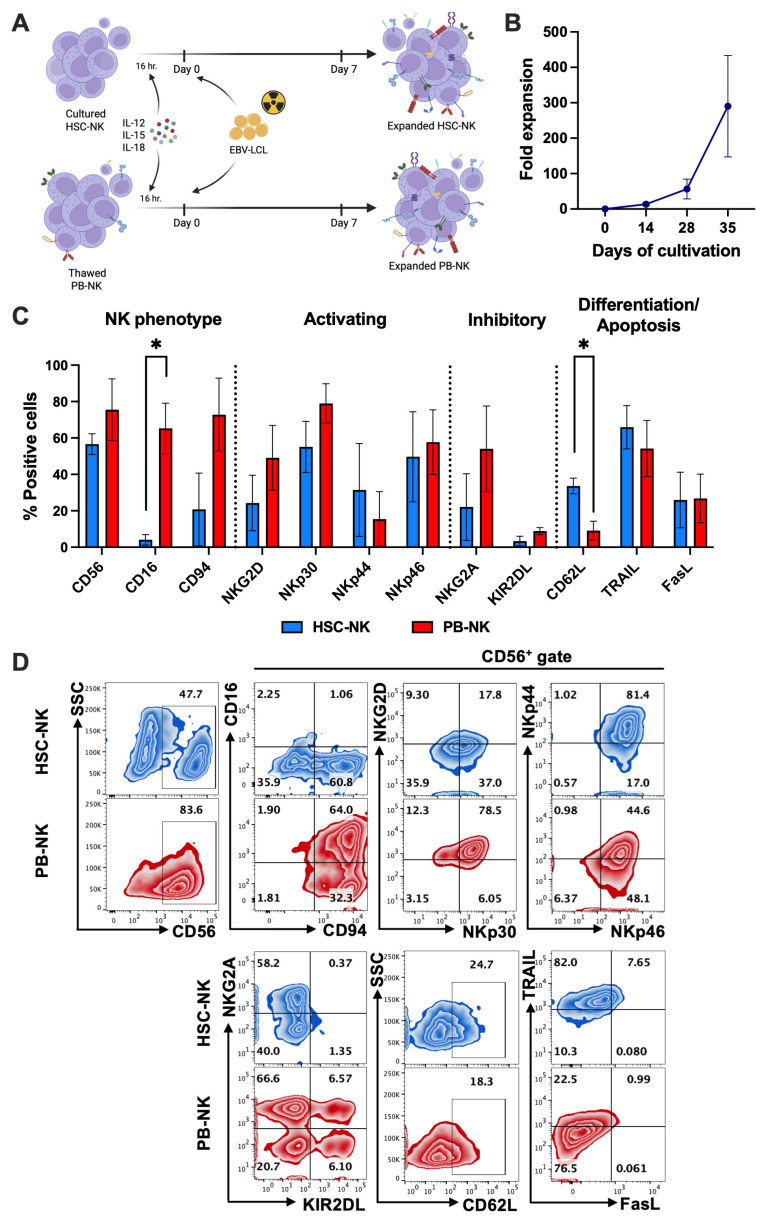
Natural killer (NK) cell expansion and immunophenotype assays. (**A**) Schematic of the NK cell expansion using cytokine-induced memory-like cocktail (interleukin-12, interleukin-15, and interleukin-18) and Epstein-Barr virus (EBV)-transformed lymphoblastoid cell line (LCL) stimulation. HSC-NK: hematopoietic stem cell-derived NK, PB-NK: peripheral blood-derived NK. (**B**) Cumulative fold expansion of HSC-derived NK during the differentiation process. (**C**) Immunophenotypes of NK cells (CD56, CD16, CD94), activating receptors (NKG2D, NKp30, NKp44, NKp46), inhibitory receptors (NKG2A, KIR2DL), differentiation marker (CD62L), and apoptosis markers (TRAIL, FasL) of HSC-NK and PB-NK cells post-expansion. (**D**) Representative flow cytometry plots of NK immunophenotypes for HSC-NK and PB-NK cells after expansion. Data are presented as the mean ± SEM and summarized from three different donors. A Student’s *t*-test was used for (**C**); * *p* < 0.01.

To enhance NK cell function, expansion, and persistence, day-28-cultivated HSC-NK cells or cryopreserved PB-NK cells were primed with a cytokine-induced memory-like cocktail (IL-12, IL-15, and IL-18) in an NK cell medium for 16 h. They were then stimulated by co-culturing with irradiated Epstein–Barr virus (EBV)-transformed lymphoblastoid cells (LCLs) at a 20:1 feeder-to-NK cell ratio for an additional 7 days (Figure 3A). Notably, HSC-NK generated from two donors–one with lymphoma (patient #2) and one with myeloma (patient #4)–yielded insufficient cell numbers to proceed with the expansion process after the initial 28 days of culture (Table S1). The accumulative HSC-NK cell expansion on days 28 and 35 reached 56-fold and 290-fold expansion, respectively (Figure 3B). Phenotypically, HSC-NK cells less expressed CD16 and CD94 compared to PB-NK cells, suggesting an immature differentiation stage (Figure 3C,D). Following stimulation, both HSC-NK and PB-NK cells showed comparable expression of NK activating receptors, including NKG2D, NKp30, NKp44, and NKp46. In contrast, HSC-NK cells exhibited a trend toward lower expression of inhibitory receptors (NKG2A and KIR2DL) compared to PB-NK cells (Figure 3C,D). Additionally, prominent expression of CD62L—which plays a critical role in NK cell homing and in vivo persistence—was observed in HSC-NK cells. Regarding apoptosis markers, both cell types showed similar levels of Tumor Necrosis Factor-Related Apoptosis-Inducing Ligand [TRAIL] and Fas Ligand [FasL] expression after expansion (Figure 3C,D). Moreover, HSC-NK cells exhibited a less exhausted phenotype compared to PB-NK cells following stimulation (Figure S1). Overall, these results demonstrate the feasibility of our differentiation and expansion protocols for generating HSC-NK cells with immunophenotypes distinct from those of PB-NK cells.

### 2.4. HSC-NK Cells Exhibit a Trend Toward an Early Activation Response to Cancer

**Figure 4:**
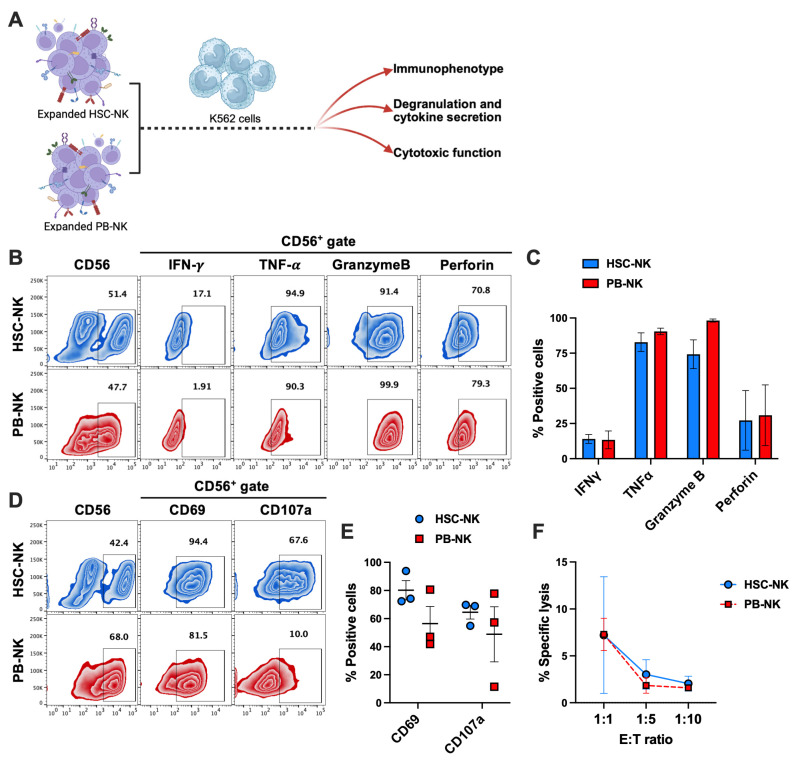
Functional assays of expanded natural killer (NK) cells. (**A**) Schematic of functional assays for expanded NK cells. HSC-NK: hematopoietic stem cell-derived NK, PB-NK: peripheral blood-derived NK. (**B**) Representative flow cytometry plots of cytokine secretion assays in NK cells post-stimulation. IFN-γ: Interferon-gamma, TNF-α: Tumor necrosis factor-alpha. (**C**) Percentages of IFN-γ, TNF-α, granzyme B, and perforin-positive NK cells following target cell stimulation. (**D**) Representative flow cytometry plots of activation and degranulation markers in NK cells post-stimulation. (**E**) Percentages of CD69 and CD107a-positive NK cells after target cell stimulation. (**F**) Specific cytolysis assay of NK cells against the K562 (chronic myeloid leukemia) cell line. CellTrace violet-labeled NK cells were incubated with tumor cells for 24 h at various E:T ratios. Dead cells were detected using 7-AAD Viability staining solution and flow cytometry. Data are pooled from three different donors and presented as the mean ± SEM. A Student’s *t*-test was used for (**C**,**E**,**F**); *p* = ns.

To mimic NK cell response to cancer, the expanded NK cells were stimulated with the K562 cell line (Figure 4A). Intracellular staining revealed that HSC-NK and PB-NK cells expressed comparable levels of IFN-γ, TNF-α, and perforin; however, PB-NK cells exhibited higher granzyme B expression (Figure 4B,C). Regarding degranulation and activation, HSC-NK cells showed a trend toward higher CD107a and CD69 expression with lower donor-to-donor variability compared to PB-NK cells, suggesting a more consistent and robust early activation response upon tumor encounter (Figure 4D,E). A specific cytolysis assay against K562 cells at various effector-to-target (E:T) ratios demonstrated comparable cytolytic activity between both NK cell types (Figure 4F). These findings suggest that HSC-NK possess early immune activation upon encountering cancer and retain strong anti-tumor properties despite their ex vivo expansion.

## 3. Discussion

Current NK cell-based immunotherapies face critical challenges, including limited donor variability, suboptimal expansion potential, and functional exhaustion of NK cells [22,23]. To overcome these hurdles, we optimized a differentiation and expansion protocol for HSC-NK cells derived from patient-derived PBSCs. This approach yielded a cumulative 290-fold expansion, resulting in HSC-NK cells with distinctive phenotypic characteristics and functional activities compared to PB-NK cells.

The archived PBSCs used in this study were obtained from five donors with varied treatment histories and heterogeneous diseases, including high-dose chemotherapy and mobilization regimens, thereby reflecting a real-world clinical cohort typically considered for autologous stem cell transplantation. Consequently, these heavily pretreated patients had required intensive chemo-mobilization regimens—such as cytarabine-based protocols or the addition of plerixafor—rather than standard G-CSF alone. We successfully generated HSC-NK cells with a 56-fold expansion from all cases; notably, three of these cases were amenable for further cultivation, reaching an average accumulative expansion of 290-fold. Previous literature indicates that bone marrow- and PB-derived HSCs typically expand by 6.5-to-690-fold [24]. While our expansion rate is substantial, it is comparatively lower than the highest rates reported in the literature. Interestingly, when comparing patients across disease types, HSC-NK cells from the youngest HL donor yielded one of the highest fold expansions. Conversely, those from heavily pretreated MM and DLBCL patients exhibited reduced proliferation, failing to yield sufficient cell numbers for the extended expansion phase. These findings suggest that rather than disease type alone, profound inter-donor variability in baseline marrow reserve and the specific cellular stress induced by distinct prior treatments—such as the incorporation of thalidomide versus cyclophosphamide—heavily dictate HSC proliferative potential, a crucial consideration when designing autologous or donor-derived cell therapies. Chemotherapy-induced hematopoietic suppression has been shown to impair progenitor cell fitness, thereby limiting ex vivo proliferation and lineage commitment. Ultimately, demonstrating robust expansion capacity from such suboptimal, chemo-mobilized, and heavily pretreated sources provides strong evidence that this platform can be effectively utilized with healthy donor HSCs to manufacture off-the-shelf CAR-NK products.

Moreover, variability in expansion efficiency is also influenced by the feeder cell type and cytokine stimulation provided during cultivation. Denman et al. demonstrated that feeder cell-based NK expansion methods utilizing membraned-bound interleukin-21 (mbIL-21) on K562 cells achieved up to a 47,967-fold expansion of PB-NK cells [25], which was attributed to IL-21 preventing NK cell senescence [26]. Alternatively, the use of γ-irradiated EBV-LCL feeder cells has been reported to induce higher expression of effector molecules—such as TRAIL, FasL, and NKG2D—and stimulate higher cytokine secretion in the generated NK cells, resulting in superior cytotoxicity against tumor cells [27,28]. Moreover, memory-like cytokine cocktail (IL-12, IL-15, and IL-18)-primed differentiated NK cells have been reported to demonstrate enhanced NK cell proliferation, robust cytotoxicity against leukemia targets, and increased IFN-γ production upon stimulation [10,11,12,29].

Similar to previous reports, we demonstrated that expanded HSC-NK cells predominantly expressed the CD56^bright^CD16^low^ subset [15]. The lower CD16 expression of HSC-NK cells represented an immature, antigen-inexperienced phenotype compared to PB-NK cells [30]. Consequently, PB-NK cells may be better suited for ADCC due to their higher CD16 expression [31]. Despite their immature differentiation, expanded CD56^bright^CD16^low^ HSC-NK cells displayed comparable activating NK cell receptors while exhibiting a trend toward lower levels of inhibitory receptors (NKG2A, KIR2DL, and CD94) relative to PB-NK cells, indicating that our differentiation and expansion protocol successfully enhanced NK functional properties. This lower inhibitory receptor expression is associated with enhanced NK cell persistence and activation in adoptive therapy settings [32], while NKG2A blockade significantly improves NK cell-mediated killing of resistant tumor cells [33].

Additionally, HSC-NK cells exhibited increased expression of CD62L, which plays a crucial role in directing NK cell differentiation capacity and homing to secondary lymphoid tissues and tumor sites [34]. Tian et al. demonstrated that the CD62L^+^ subset is required for NKT ex vivo expansion and in vivo persistence; furthermore, engineered CD62L^+^ CD19CAR NKT cells produced sustained tumor regression in a murine model of B-cell lymphoma [35]. Although TRAIL-mediated signaling contributes to tumor cell apoptosis via death receptor pathways [36], TRAIL was not significantly expressed in EBV-LCL-expanded HSC-NK cells. These phenotypic traits suggest that our expanded HSC-NK cells may have an advantage in overcoming immune evasion, making them a promising candidate for immunotherapy targeting tumors that exhibit strong immune resistance.

Functional assays further validated the cytotoxic potential of HSC-NK cells, revealing consistently elevated levels of the activation and degranulation marker; CD69 and CD107a, which play a direct role in mediating cytotoxicity against tumor targets [37,38]. Most importantly, direct co-culture assays demonstrated that HSC-NK cells executed specific cytolysis against K562 cancer cells at levels highly comparable to conventional PB-NK cells. These comprehensive functional data confirm that despite exhibiting an immature surface phenotype with lower CD16 expression, expanded HSC-NK cells retain the potent degranulation and anti-tumor cytotoxicity capabilities essential for adoptive immunotherapy, demonstrating that their generated phenotype successfully translates into effective functional activity.

Regarding the optimal timing for the collection of mature NK cells following ASCT, Clausen et al. demonstrated that the proliferative capacity of CD56^+^ cells obtained from PBSC harvests and after ASCT is impaired compared to those collected one week post-ASCT [39]. Moreover, another study by Clausen and colleagues showed that CD34^+^ cells and T-cells have an inhibitory effect on NK-cell proliferation during PBSC mobilization with G-CSF [40]. In this study, we aimed to assess the feasibility of generating HSC-NK cells and to compare their therapeutic potential to that of conventional PB-NK cells. Therefore, we collected the PBSC for further cultivation and separated PB-NK at the same timepoint. Nevertheless, our data strongly suggest that our intense ex vivo culture conditions effectively overcome the aforementioned limitations. Moreover, in our current small-scale in vitro model, the cryopreservation of the baseline PB-NK cells was an unavoidable logistical necessity to synchronize their expansion and functional assays with the 28-day differentiation cycle of the HSC-NK cells.

The primary limitation of this study is the small sample size, which was dictated by the availability of sufficient cell numbers for differentiation and expansion; however, our findings still demonstrate inter-donor variability in the process of HSC-NK cell differentiation. Furthermore, the exact absolute number of HSC-NK cells required for optimal clinical dosing remains to be definitively established. Our data demonstrated specific cytolysis against tumor cells even at an E:T ratio of 1:10. This robust in vitro potency suggests high per-cell efficacy; however, further in vivo testing and the transition to large-scale clinical manufacturing platforms are necessary to determine if sufficient therapeutic doses can be consistently achieved. Another critical consideration is the in vivo persistence and tumor-homing efficiency of these expanded HSC-NK cells.

Another critical consideration is the in vivo persistence and tumor-homing efficiency of expanded HSC-NK cells. While our phenotypic findings suggest that HSC-NK cells may have an advantage in overcoming immune evasion, further research is needed to assess whether these traits translate into prolonged in vivo efficacy. Additionally, while the feeder-free, cytokine-based expansion system offers significant advantages over feeder-dependent approaches [24], future work should further refine cytokine cocktails and metabolic support strategies to enhance NK cell function. Given the growing interest in genetically modified NK cells, future research should also explore engineered HSC-NK cells—including CAR-NK strategies—to enhance tumor specificity and resistance to immune evasion mechanisms.

## 4. Materials and Methods

### 4.1. Cell Lines

The human cell lines, K562 and EBV-LCL, utilized in this study were originally obtained from Dr. Seitaro Terakura (Department of Hematology and Oncology, Nagoya University Graduate School of Medicine, Nagoya, Japan). The K562 cell line has been maintained in his laboratory, while the EBV-LCL cell line was generated in-house by his research team. K562 and EBV- LCL were maintained in our laboratory and cultured in RPMI-1640 (Gibco, Life Technologies Corp., Carlsbad, CA, USA) supplemented with 10% fetal bovine serum (FBS), 1% penicillin–streptomycin (PS), and 0.8 mM L-glutamine (LG). Standard culture conditions were used: 37 °C in a humidified atmosphere containing 5% CO_2_.

### 4.2. Human Samples

PBSC apheresis products were obtained from patients undergoing HSCT at Songklanagarind Hospital. All participants provided written informed consent in accordance with the Declaration of Helsinki, under a protocol approved by the Institutional Review Board-approved protocol at the Faculty of Medicine, Prince of Songkla University.

### 4.3. Cell Isolation

MNCs were isolated from the apheresis products using Lymphoprep^TM^ density gradient centrifugation (STEMCELL Technologies Inc., Vancouver, BC, Canada). The isolated MNCs were then incubated with anti-human CD34^+^ or anti-human CD56^+^ immunomagnetic beads (Miltenyi Biotec, Bergisch Gladbach, Germany) and subsequently enriched through magnetic columns. The purity of the enriched CD34^+^ PBSC and CD56^+^ PB-NK cell populations was analyzed by flow cytometry. The isolated PBSCs underwent a differentiation and expansion process to generate HSC-NK cells, while the PB-NK cells were cryopreserved for later comparative experiments (Figure 1A).

### 4.4. HSC-NK Cell Generation

The enriched CD34^+^ cells were cultured in a two-step consisting of a lymphoid progenitor differentiation phase (days 0–14) followed by an NK cell differentiation (days 14–28) (Figure 1A). Initially, 2.5 × 10^4^ enriched CD34^+^ cells were cultivated in StemSpan™ SFEM II medium supplemented with StemSpan™Lymphoid Progenitor Expansion Supplement (STEMCELL™ Technologies, Vancouver, BC, Canada), which SCF, TPO, Flt3L, and IL-7. Cells were seeded onto 24-well non-tissue culture plates pre-coated with StemSpan™ Lymphoid Differentiation Coating Material. On day 14, the differentiated lymphoid progenitor cells were harvested and analyzed by flow cytometry to identify lymphoid progenitors using the markers CD45, CD34, CD5, and CD7. Cells expressing CD5 and/or CD7 were classified as early lymphoid progenitors, counted, and re-plated at 5 × 10^4^ cells onto new pre-coated 24-well plates. The cells were subsequently cultured in StemSpan^TM^ NK Cell Differentiation Medium for an additional 14 days. On day 28, the differentiated CD56^+^ HSC-NK cells were harvested, counted, and analyzed. At each stage of HSC-NK cell generation, cell aliquots were assessed for immunophenotypic profiles by flow cytometry. An illustration of the HSC-NK cell generation protocol is shown in Figure 2A.

### 4.5. NK Cell Expansion

The HSC-NK or PB-NK cells were primed with a cytokine-induced memory-like cocktail (containing 10 ng/mL IL-12, 1 ng/mL IL-15, and 50 ng/mL IL-18) [29] and cultured for 16 h in NK cell medium (RPMI-1640 supplemented with 10% human serum, 2% L-glutamine, 1% penicillin–streptomycin, 200 IU/mL IL-2, and 0.5 μM 2-mercaptoethanol). The following day, the primed NK cells were washed and stimulated via co-culture with irradiated EBV-LCLs at a 20:1 feeder-to-NK cell ratio for an additional 7 days. The expanded NK cells were subsequently harvested, counted, and used for immunophenotyping and downstream assays. The absolute number of NK cells was calculated by multiplying the total viable cell count by the percentage of CD56^+^/CD3^−^ cells determined by flow cytometry. To evaluate the overall efficiency of the generation process, the cumulative fold expansion of HSC-NK cells was calculated relative to the number of initially seeded CD34^+^ cells on day 0. This cumulative metric was determined for each donor by multiplying the individual fold expansions achieved during each phase of the culture process to account for the sequential re-plating of specific cell aliquots.

### 4.6. NK Cell Immunophenotype Assay

To characterize the various stages of NK cell differentiation, HSC-NK or PB-NK cells were immunophenotypically stained with monoclonal antibodies and analyzed by flow cytometry. On day 0, an anti-CD34 monoclonal antibody was used to identify HSCs and CD56 was used for NK cells. For the identification of lymphoid progenitor cells on day 14, HSC-differentiated cells were stained with anti-CD45, CD3, CD5, CD7, and CD56 monoclonal antibodies. Following NK cell differentiation (day 28), cells were assessed for mature NK cell phenotypes using a panel that included CD45, CD3, CD16, CD56, and CD94. After expansion, the NK cells were stained with the following panels: (1) activating receptors (NKp30, NKp44, NKp46, and NKG2D); (2) inhibitory receptors (KIR2DL and NKG2A); (3) the homing/differentiation marker CD62L; (4) apoptotic markers (TRAIL and FasL); and (5) exhaustion markers (PD-1, CTLA-4, TIM-3, and LAG3) (BioLegend, San Diego, CA, USA). All samples were analyzed using a BD FACSAria^TM^ Fusion (BD Biosciences, Franklin Lakes, NJ, USA), and data were processed using FlowJo software version 11.0.2 (Tree Star, Ashland, OR, USA).

### 4.7. Degranulation and Cytokine Secretion Assay

The expanded HSC-NK or PB-NK cells were stimulated with K562 target cells at an E:T ratio of 2:1 ratio for 4 h. After 2 h, GolgiPlug™ Protein Transport Inhibitor (BD Biosciences, Franklin Lakes, NJ, USA) was added, and cells were incubated for an additional 2 h to allow intracellular cytokine accumulation. The cells were then harvested and stained for degranulation and activation using CD107a and CD69 monoclonal antibodies (BioLegend, San Diego, CA, USA) on the CD56^+^/CD3^−^ cell population. Subsequently, the cells were fixed and permeabilized using Cytofix/Cytoperm™ Fixation/Permeabilization Kit (BD Bioscience, Franklin Lakes, NJ, USA) and stained intracellularly for granzyme B, perforin, IFN-γ, and TNF-α (BD Bioscience, Franklin Lakes, NJ, USA). All samples were analyzed via flow cytometry.

### 4.8. NK Cell Cytotoxicity Assay

To investigate NK cell-mediated cytolysis, HSC-NK or PB-NK cells were pre-stained with CellTrace™ Violet (CTV; Thermo Fisher Scientific, Waltham, MA, USA) and then co-cultured with K562 cells at different E:T ratios of 1:1, 1:5, and 1:10 in 96-well plates in NK cell medium for 24 h. The cells were subsequently stained with 7-Amioactinomycin D (7-AAD) to identify dead or apoptotic cells and analyzed by flow cytometry. Target cells were gated on the CTV-negative population. The percentage of specific cytolysis was calculated using the following formula: [(target cell death − spontaneous cell death)/(100 − spontaneous cell death)] × 100.

### 4.9. Statistical Analysis

All experimental data are presented as the mean ± standard error of the mean (SEM). Differences were considered statistically significant at *p* < 0.05. Flow cytometry data were analyzed using FlowJo software (Tree Star, Ashland, OR, USA), and all statistical analyses were conducted using GraphPad Prism (Version 11.0.0 software, GraphPad Software, La Jolla, CA, USA).

## 5. Conclusions

This study demonstrated the successful cultivation of NK cells from mobilized PBSCs obtained from patients with heavily pretreated hematologic malignancies. Superior expansion yields were observed with younger donor age, reflecting preserved progenitor fitness and highlighting the critical impact of prior therapy on manufacturing feasibility. Despite heterogeneity in expansion yields, the functional properties of HSC-NK cells remained consistently high across all donors, translating into potent anti-tumor activity against leukemic targets.

In summary, generating functional, reprogrammed HSC-NK cells is a feasible strategy, offering a promising alternative immune cell source for cancer immunotherapy.

## Data Availability

The original contributions presented in this study are included in the article/Supplementary Material. Further inquires can be directed to the correspponding author.

## Supplementary Materials

The following supporting information can be downloaded at: https://www.mdpi.com/article/10.3390/ijms27135836/s1.
