# Supplementary material for: Cultivating Functional Natural Killer Cells from Mobilized Hematopoietic Stem Cells in Heavily Pretreated Hematologic Malignancies
Source: Int J Mol Sci. 2026 Jun 28;27(13):5836. doi: 10.3390/ijms27135836 (PMC13361641; doi:10.3390/ijms27135836)
Supplement: Supplementary file 1 [file ijms-27-05836-s001.zip › ijms-4365062-supplementary.pdf]

Supplementary Material

Table S1: Summary results of individual HSC-derived NK cell generation.

No	Diagnosis	Age (year) /Sex	Chemotherapy (cycles)/ Mobilizing agents	Immunophenotype		Day 35 HSC-NK fold expansion
				Day 14 Progenitor T-cell ($CD5^+/CD7^+$)	Day 28 Mature NK-cell ($CD56^+$)	
1	MM	66/M	VCD (6) /HD CY + G-CSF	30%	45%	401.12
2	DLBCL	48/M	R-CHOP (6), ICE (2), DHAP (3) /DHAP + G-CSF	50%	28%	15.3 (Day 28)
3	HL	37/M	ABVD (2), BV-DHAP (4) /HD CY + Plerixafor + G-CSF	82%	57%	463.59
4	MM	65/F	VTD (6) /HD CY + G-CSF	20%	50%	33.4 (Day 28)
5	MM	66/M	VTD (5) /HD CY + G-CSF	20%	60%	6.3

HSC, hematopoietic stem cell; NK, natural killer; DLBCL, diffuse large B-cell lymphoma; HL, Hodgkin lymphoma; MM, multiple myeloma; F, female; M, male; VCD, bortezomib-cyclophosphamide-dexamethasone; HD CY, high-dose cyclophosphamide; G-CSF, granulocyte colony- stimulating factor; R-CHOP, rituximab-doxorubicin-cyclophosphamide-vincristine-prednisolone; ICE, ifosfamide-carboplatin-etoposide; DHAP, dexamethasone-cisplatin-cytarabine; ABVD, doxorubicin-bleomycin-vinblastine-dacarbazine; BV, Brentuximab vedotin; VTD, bortezomib-thalidomide-dexamethasone.

Figure S1

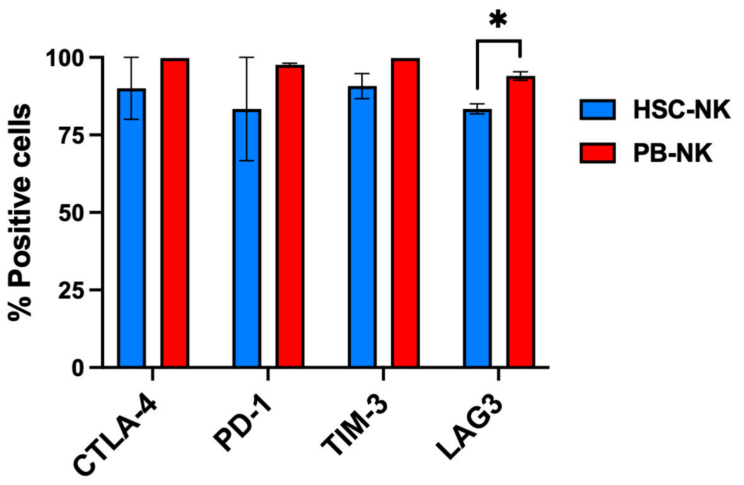

Natural killer (NK) cell exhaustion immunophenotypes. Immunophenotypes of expanded NK cell (day 35); CTLA-4: cytotoxic T-lymphocyte-associated protein 4, PD-1: programmed death protein 1, TIM-3: T-cell immunoglobulin and mucin-domain containing-3, and LAG3: lymphocyte-activation gene 3. Data are presented as mean ± SEM and summarized from two different donors; Student's *t*-test was used; *$p < 0.01$.
